# Modulation of Brain Functional Connectivity and Efficiency During an Endurance Cycling Task: A Source-Level EEG and Graph Theory Approach

**DOI:** 10.3389/fnhum.2020.00243

**Published:** 2020-07-09

**Authors:** Gabriella Tamburro, Selenia di Fronso, Claudio Robazza, Maurizio Bertollo, Silvia Comani

**Affiliations:** ^1^Behavioral Imaging and Neural Dynamics Center, University “G. d’Annunzio” of Chieti–Pescara, Chieti, Italy; ^2^Department of Neurosciences, Imaging and Clinical Sciences, University “G. d’Annunzio” of Chieti–Pescara, Chieti, Italy; ^3^Department of Medicine and Aging Sciences, University “G. d’Annunzio” of Chieti–Pescara, Chieti, Italy

**Keywords:** EEG, cycling, functional connectivity, source level, Graph Theory, efficiency, endurance task

## Abstract

Various methods have been employed to investigate different aspects of brain activity modulation related to the performance of a cycling task. In our study, we examined how functional connectivity and brain network efficiency varied during an endurance cycling task. For this purpose, we reconstructed EEG signals at source level: we computed current densities in 28 anatomical regions of interest (ROIs) through the eLORETA algorithm, and then we calculated the lagged coherence of the 28 current density signals to define the adjacency matrix. To quantify changes of functional network efficiency during an exhaustive cycling task, we computed three graph theoretical indices: local efficiency (LE), global efficiency (GE), and density (D) in two different frequency bands, Alpha and Beta bands, that indicate alertness processes and motor binding/fatigue, respectively. LE is a measure of functional segregation that quantifies the ability of a network to exchange information locally. GE is a measure of functional integration that quantifies the ability of a network to exchange information globally. D is a global measure of connectivity that describes the extent of connectivity in a network. This analysis was conducted for six different task intervals: pre-cycling; initial, intermediate, and final stages of cycling; and active recovery and passive recovery. Fourteen participants performed an incremental cycling task with simultaneous EEG recording and rated perceived exertion monitoring to detect the participants’ exhaustion. LE remained constant during the endurance cycling task in both bands. Therefore, we speculate that fatigue processes did not affect the segregated neural processing. We observed an increase of GE in the Alpha band only during cycling, which could be due to greater alertness processes and preparedness to stimuli during exercise. Conversely, although D did not change significantly over time in the Alpha band, its general reduction in the Beta bands during cycling could be interpreted within the framework of the neural efficiency hypothesis, which posits a reduced neural activity for expert/automated performances. We argue that the use of graph theoretical indices represents a clear methodological advancement in studying endurance performance.

## Introduction

Studies performed in the last decade have assessed that cycling induces specific changes in brain cortical activity (Schneider et al., 2009, 2013; Ludyga et al., 2016; Bao et al., 2019). These changes can be measured by electroencephalography (EEG), and various methods have been employed to investigate different aspects of modulation in regional brain activity related to the performance of a cycling task.

For instance, Maceri et al. (2019) focused on the variations of signal power (quantified by means of power spectral density—PSD) in the prefrontal and the motor cortex during an incremental cycling test and found a significant increase of PSD in the Alpha and Beta bands in association with an increase of exercise intensity, particularly during the last two stages of the incremental test. Other studies employed independent component analysis (ICA) and models of signal sources to identify the brain areas maximally involved in a cycling task, such as in the study by Enders et al. (2015), who applied ICA and an equivalent current dipole model to the EEG signals recorded during a time-to-exhaustion test. They identified four cortical areas (two in the parietal cortex and two in the frontal cortex) showing a significant increase in signal power, which is likely due to fatigue developed during the exercise. On the other hand, Brümmer et al. (2011) used the low-resolution brain electromagnetic tomography (LORETA) technique to analyze low-resolution EEG data and to investigate the effect of increasing cycling intensity on the magnitude and the location of the changes in electrocortical current density, showing that current density in the primary motor cortex increases with increasing exercise intensity. With a similar approach, Schneider et al. (2013) studied how motor cortex activity varied during a moderate- to high-intensity cycling exercise.

Other studies go a step further and calculate functional connectivity patterns within the brain, with the purpose to identify the routes of information flow during the different phases of a cycling task. For instance, Comani et al. (2014) analyzed EEG data recorded during an exhaustive cycling test by calculating the coherence patterns at the sensor level and detected functional connectivity patterns related to three different attentional strategies. Similarly, di Fronso et al. (2018) performed a coherence analysis to detect cortical connectivity patterns in relation to the attentional strategies and the phases of an endurance cycling task. The Alpha and Beta bands were considered, and the results showed higher EEG coherence values at rest than during cycling periods for all electrode pairs and frequency bands, irrespective of the attentional strategy adopted. Hilty et al. (2011) also performed a functional connectivity study on EEG data recorded during a fatiguing cycling exercise to investigate the intracortical communication between the mid/anterior insular and the motor cortex. They evaluated the lagged phase synchronization (LPS) (Pascual-Marqui, 2007a) between these two specific brain areas and found that LPS significantly increased at the end of the cycling task and returned to baseline after the participants volitionally stopped the exercise, hence demonstrating that the mid/anterior insular cortex communicates with the motor cortex during a muscle-fatiguing activity.

Coherence and lagged phase synchronization permit to reconstruct networks of spatially distributed electrodes (sensor level) or brain areas (source level) that are functionally connected during the performance of a given task. These networks provide an overview of the functional interactions between neighboring and distant brain regions (Friston, 2011) but do not elucidate how information is exchanged within the functional network and do not identify its functional properties—such as the type of efficiency. A Graph Theory perspective can be beneficial in this regard (Latora and Marchiori, 2001). According to a graph theoretical approach, the brain is defined as a network containing nodes and edges, which respectively, represent brain regions and the connecting pathways between those regions (Bullmore and Sporns, 2009; Rubinov and Sporns, 2010). Very recently, Porter et al. (2019) used EEG electrodes space graph theoretical analysis to examine Theta band functional connectivity during a combined graded working memory and exercise task on a stationary bicycle. Several graph theoretical indices were computed, and the analysis revealed significant changes only for the clustering coefficient, which is a measure of the prevalence of local connectivity within a functional network. Specifically, Porter et al. (2019) found that local connectivity increased during the first three blocks of the exercise and decreased toward the end of the exercise, when the task became more difficult. However, these results are limited because the graph theory analysis was applied to the frontal region only: as a consequence, the overall properties of the complete functional brain network underpinning the cycling task remained unexplored. Further studies should examine the properties of functional networks over the whole brain.

Drawing on the aforementioned research results, we conducted a within-subject study to examine how functional connectivity and brain network efficiency varied during the performance of an endurance cycling task. For this purpose, we reconstructed the EEG signals at the source level by using the extended Loreta (eLoreta) algorithm (Pascual-Marqui et al., 2018b), calculated lagged coherence maps (Pascual-Marqui, 2007a, b; Pascual-Marqui et al., 2011, 2018a), and computed three graph theoretical indices—namely, local efficiency (LE), global efficiency (GE), and density (D)—to examine local and global functional properties. LE is a measure of functional segregation that quantifies the ability of a network to exchange information locally, GE is a measure of functional integration that quantifies the ability of a network to exchange information globally, and D is a global measure of connectivity that describes the extent of connectivity in a network (Sporns et al., 2004; Bullmore and Sporns, 2009; Rubinov and Sporns, 2010; Friston, 2011). All measures were computed for both Alpha (8–13 Hz) and Beta (13–20 Hz) frequency bands because Alpha activity is associated with alertness level (Wilson et al., 2011), whereas Beta activity provides information on motor binding and fatigue (Laufs et al., 2003; Cheron et al., 2016). Given the scant research that applies graph theoretical indices on endurance performance, the current investigation can be considered exploratory in nature.

## Materials and Methods

### Participants

Fourteen male volunteers (26.07 ± 4.29 years) participated in the study. They regularly practiced cycling at least twice a week, did not report neurological, psychological, or dermatological diseases, and were not under pharmacological treatment. The study was approved by the Ethics Committee of the University “G. d’Annunzio” of Chieti-Pescara (Italy) (ethical approval Ref. No. 10-21/05/2015) and complied with the ethical standards outlined in the Declaration of Helsinki. Prior to study participation, all the volunteers provided medical certification of fitness for participation to non-competitive sports activities and gave written informed consent according to the policies outlined. The participants were instructed to not engage in vigorous exercise and to avoid consuming alcohol, caffeinated drinks/foods, or ergogenic substances for at least 24 h prior to data collection.

### Measurement Setup

The EEG signals were continuously recorded with a conventional gel-based cap (Waveguard original, ANT Neuro) with 64 AgCl electrodes in a layout based on the extended international 10–20 system for electrode placement (Jurcak et al., 2007). CPz and AFz were used as reference and ground, respectively. Nasion, inion, and preauricular points were used as anatomical landmarks to position the EEG cap. Conductive gel for electrophysiological measurements was used (OneStep Cleargel, H+H Medizinprodukte), and impedance was kept below 10 kΩ (6.11 ± 2.18 kΩ) to comply with current standards in cognitive neurosciences. The EEG cap was connected to an EEG mobile amplifier (eego sports, ANT Neuro b. v., Hengelo, Netherlands), and the sampling rate was 1,024 samples/s, using the corresponding eego software (ANT Neuro b. v., Hengelo, Netherlands). A Monark Cycle-Ergometer (939 E, Monark Exercise AB, Vansbro, Sweden), power-controlled by an external device (Fitmate-PRO, Cosmed, Rome, Italy), was used for the endurance cycling task. Two qualified researchers collected the data. The data collection occurred in a quiet and safe environment to guarantee the participants’ comfort.

### Endurance Cycling Task

The task started from the instant when the volunteer sat on the cycle-ergometer. After a pre-cycling EEG recording period of 2 min with eyes closed and no movement, participants performed an endurance cycling task (graded exercise test using a ramp protocol) until exhaustion. We chose an exhaustive task because we intended to observe also functional connectivity patterns during post-exhaustion recovery. Participants were instructed to maintain a constant pedaling rate of approximately 80 revolutions per minute, and the power of the cycle-ergometer, initially set at 50 W, was increased by 25 W every 2 min. Rated perceived exertion was collected every minute during cycling to detect the participants’ exhaustion using the CR-10 Borg scale (Borg and Borg, 2010; Staiano et al., 2018). Given that reaching exhaustion is an individual process that depends on the participants’ fitness and expertise (di Fronso et al., 2018, 2019), the duration of the endurance task ranged from approximately 10 to 28 min (18 ± 5 min). Exhaustion was immediately followed by an active recovery period of 2 min, during which the participants continued cycling with eyes closed and the ergometer power was set back to 50 W. The task ended with a passive recovery period of 2 min, during which the participants were asked to stop cycling and maintain their eyes closed while sitting on the cycle-ergometer.

### Data Analysis

Data analysis consisted in four main steps: EEG data preprocessing, reconstruction of source EEG signals, EEG functional connectivity estimation, and calculation of measures of functional network properties.

#### EEG Data Preprocessing

The EEG signals were visually inspected by two trained EEG experts to identify bad channels (i.e., channels exhibiting either a saturated, isoelectric line or a predominantly artifactual, non-physiological signal for more than 50% of the EEG recording), which were excluded from further processing. The retained EEG traces were band-pass filtered from 3 to 40 Hz through zero-phase Hamming-windowed sinc FIR filters applied using the firfilt EEGLAB plugin (Widmann et al., 2015). The filtered EEG signals were epoched based on the task structure. Four task periods were identified: pre-cycling, cycling, and active and passive recovery. Given the different duration of the cycling period across participants, for each participant, it was divided in three stages, each including one third of the entire cycling period, thus allowing us to compare cycling periods of different durations (i.e., across participants). Therefore, for each participant, we obtained a total of six intervals: pre-cycling, cycling stage 1, cycling stage 2, cycling stage 3, active recovery, and passive recovery. For each interval, the filtered EEG data were processed with a principal component analysis for dimension reduction and ICA (ICA-extended infomax algorithm in the EEGlab toolbox) (Bell and Sejnowski, 1995; Lee et al., 1999; Delorme and Makeig, 2004) to obtain independent signal components (ICs). ICs containing eye blinks, eye movements, myogenic, movement, and environmental artifacts were disregarded, and the remaining ICs were re-projected onto the electrode space to reconstruct de-noised EEG source signals. The EEG signals at the sites of the removed bad channels were reconstructed by interpolating neighboring signals using the spherical spline interpolation feature implemented in the EEGLab toolbox (Perrin et al., 1989). The signals from 13 electrodes located in the peripheral and the occipital areas of the scalp (FT7, T7, TP7, P7, PO7, O1, Oz, O2, PO8, P8, TP8, T8, and FT8) were disregarded because of residual myogenic and movement artifacts. Consequently, given that mastoid electrodes are generally not used, we kept 49 out of 64 EEG signals for further analysis. Preprocessing was performed using EEGlab release 13.3.2b and some functions developed using Matlab (release MatlabR2016b; Mathworks, Natick, MA, United States).

#### Reconstruction of Source EEG Signals

**FIGURE 1:**
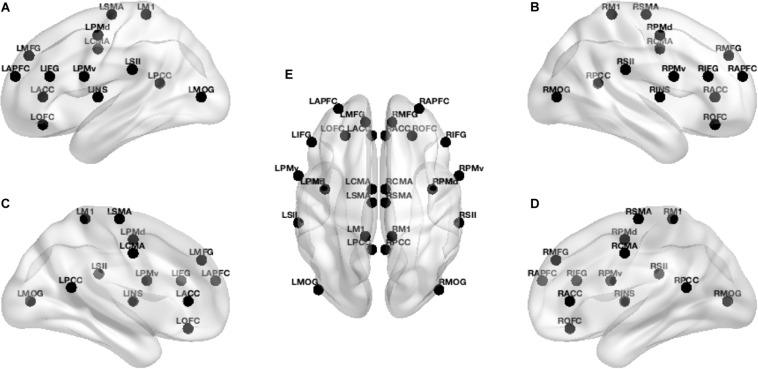
Localization of the 28 ROIs in the brain. From left to right: **(A,C)** lateral and medial left view of the brain, **(E)** dorsal view of the brain, and **(B,D)** lateral and medial right view of the brain. In **(E)**, view from front to back: L/R-APFC (anterior prefrontal cortex), L/R-MFG (middle frontal gyrus), L/R-ACC (anterior cingulate cortex), L/R-OFC (orbitofrontal cortex), L/R-IFG (inferior frontal gyrus), L/R-PMv (ventral premotor cortex), L/R-PMd (dorsal premotor cortex), L/R-CMA (cingulate motor area), L/R-INS (insula), L/R-SMA (supplementary motor area), L/R-SII (secondary sensorimotor area), L/R-M1 (primary motor cortex), L/R-PCC (posterior cingulate cortex), L/R-MOG (middle occipital gyrus), where L is for the left and R is for the right regions of interest.

Grounded on previous studies (Christensen et al., 2000; Sahyoun et al., 2004; Ciccarelli et al., 2005; Mayka et al., 2006; Francis et al., 2009; Mehta et al., 2009, 2012; Gandolla et al., 2014; Jaeger et al., 2014) and according to the realistic head model employing Montreal Neurophysiological Institute 152 template (Mazziotta et al., 2001a, b), we defined 14 regions of interest (ROIs) (see Table 1) for each hemisphere to provide a broad coverage of brain areas (see Figure 1) possibly active during movement of the lower limbs. Based on the scalp-recorded electric potential distribution, eLORETA (Pascual-Marqui et al., 2018b) algorithm (LORETA KEY-software^1^) was used to compute the cortical three-dimensional distribution of current density for each ROI and each task period. The eLORETA algorithm is a linear inverse solution for EEG signals that has no localization error to point sources under ideal (noise-free) conditions (Pascual-Marqui, 2002).

**TABLE 1:** Montreal Neurophysiological Institute (MNI) coordinates of the 28 regions of interest (ROIs) used to analyze the electroencephalograph signal of each cyclist.

Label	Extended label	Brodmann area	Hemisphere	ROI (MNI coordinates)		
				X	Y	Z
APFC	Anterior pre-frontal cortex	10	Left	−30	55	20
			Right	30	55	20
MFG	Middle frontal gyrus	9	Left	−10	45	35
			Right	10	45	35
ACC	Anterior cingulate cortex	24	Left	−5	35	5
			Right	5	35	5
OFC	Orbitofrontal cortex	11	Left	−25	35	−15
			Right	25	35	−15
IFG	Inferior frontal gyrus	46	Left	−50	30	20
			Right	50	30	20
PMv	Ventral pre-motor area	44	Left	−60	5	20
			Right	60	5	20
PMd	Dorsal pre-motor area	6	Left	−40	−5	50
			Right	40	−5	50
CMA	Cingulate motor area	24	Left	−5	−5	40
			Right	5	−5	40
INS	Insula	13	Left	−40	−5	5
			Right	40	−5	5
SMA	Supplementary motor area	6	Left	−5	−15	65
			Right	5	−15	65
SII	Secondary sensory motor area	40	Left	−60	−30	25
			Right	60	−30	25
M1	Primary motor area	4a	Left	−10	−40	65
			Right	10	−40	65
PCC	Posterior cingulate cortex	30	Left	−5	−50	15
			Right	5	−50	15
MOG	Middle occipital gyrus	19	Left	−45	−80	5
			Right	45	−80	5

#### EEG Functional Connectivity Estimation

Coherence is usually considered as a good estimator of functional connectivity between electrodes/sources. Based on the method described by Pascual-Marqui (2007a, b); Pascual-Marqui et al. (2011, 2018a), we estimated functional connectivity maps across the 28 ROIs by calculating the lagged coherence (LC) for each task interval in the Alpha (8–12 Hz) and Beta (12–30 Hz) bands. We used LC because it is less affected by volume conduction effects (Pascual-Marqui et al., 2018a). For each frequency band and each participant, we then obtained six matrices of LC values that could range between 0 and 1, where 0 indicates no coherence and 1 indicates perfect coherence between two time series of current density. To retain only significant functional connections across ROIs, we thresholded the LC matrices (Berchicci et al., 2015; di Fronso et al., 2018): We calculated the median and the median absolute deviation (MAD) of the LC value distribution for each LC matrix; only LC values > (median + 1 MAD) were considered as meaningful functional connections, retained and set equal to 1, whereas all other LC values were set equal to 0. Therefore, we obtained 12 (28 × 28) undirected binary adjacency matrices for each subject (six matrices for each frequency band). Functions used to estimate EEG functional connectivity were developed using Matlab (release MatlabR2016b; Mathworks, Natick, MA, United States).

**FIGURE 2:**
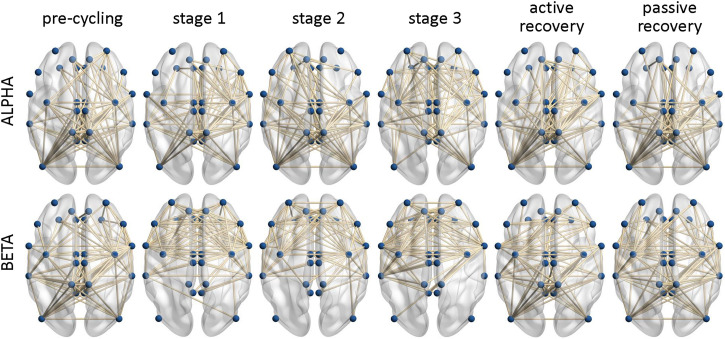
Binary functional connectivity maps averaged over all participants. The maps are calculated for the six task intervals (pre-cycling, cycling stage 1, cycling stage 2, cycling stage 3, active recovery, and passive recovery) in the Alpha and the Beta frequency bands. The labels of the individual brain areas were omitted for clarity.

Figure 2 shows the undirected binary adjacency matrices averaged over all participants for each frequency band and each task interval.

#### Measures of Functional Network Properties

We applied graph theoretical analysis to typify the organization of the functional networks represented by undirected binary adjacency matrices. For this purpose, we calculated the following graph theoretical indices: LE, GE, and average connection D. LE is a measure of functional segregation calculated as the global efficiency on the neighborhood of a node, and it quantifies the ability of a network to exchange information locally. A brain network is functionally segregated when the information processing occurs within densely interconnected groups of neighboring brain areas (Rubinov and Sporns, 2010). GE is a measure of functional integration (Rubinov and Sporns, 2010; Friston, 2011) defined as the average inverse shortest path length in the network (Sporns et al., 2004; Bullmore and Sporns, 2009). GE quantifies the ability of a network to exchange information globally. Specifically, a brain network is functionally integrated when it combines specialized information exchange from distributed brain regions (Rubinov and Sporns, 2010; Friston, 2011). D is a global measure of connectivity that describes the extent of connectivity in a network (Bullmore and Sporns, 2009; Bassett and Bullmore, 2017). It is defined as the number of actual connections divided by the number of all possible connections in the graph. D varies from 0 to 1: the sparser the graph, the lower is D. Overall calculations of GE, LE, and D were performed using the Brain Connectivity Toolbox (Rubinov and Sporns, 2010).

#### Statistical Analysis

The distributions of the values obtained for GE, LE, and D were Gaussian. Hence, we performed a series of one-way analysis of variance (ANOVA) using Bonferroni correction for *post hoc* pairwise comparisons to statistically compare the values of the three graph metrics over the six task intervals for each frequency band. The sphericity assumption was evaluated using the Mauchly test. Greenhouse–Geisser and Huynh–Feldt corrections for degrees of freedom were applied in case of non-sphericity. In the analysis of variance, the effect sizes were calculated using partial eta square (η_p_^2^) (Lakens, 2013), for which 0.01, 0.06, and 0.14 were considered as small, medium, and large effects, respectively. In the case of multiple comparisons, the effect sizes were calculated using Cohen’s *d* (Cohen, 1988; Lakens, 2013), for which 0.20, 0.50, and 0.80 were considered as small, medium and large effects, respectively. For each computed ANOVA, the significance level was set at 0.05. Statistical analysis was performed using the Statistical Package for Social Sciences software (SPSS v. 25, IBM, Armonk, United States).

## Results

**FIGURE 3:**
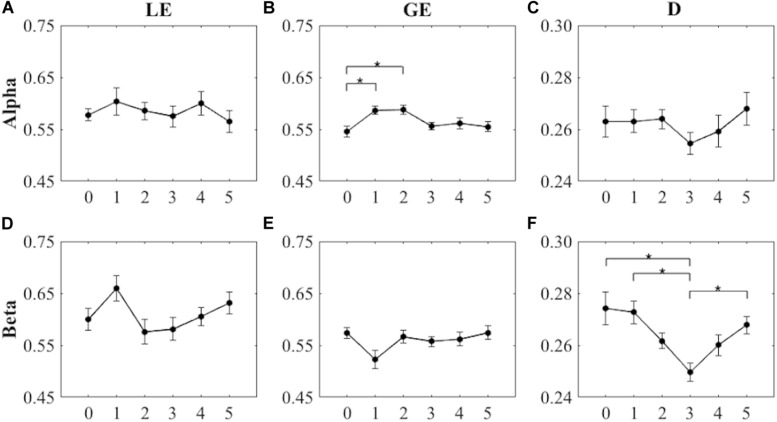
Values of the graph theoretical indices (LE, local efficiency; GE, global efficiency; D, density) over the six task intervals in the Alpha and the Beta bands (asterisks indicate significant differences between the different intervals; *p* < 0.05). **(A,D)** Local efficiency (mean ± SEM). **(B,E)** Global efficiency (mean ± SEM). **(C,F)** Density (mean ± SEM). Error bars represent SEM.

As shown in Figures 3A,D, the within-subjects one-way ANOVA for LE values did not show significant differences among the six intervals (i.e., pre-cycling, cycling stage 1, cycling stage 2, cycling stage 3, and active and passive recovery) of the endurance cycling task for the two frequency bands [Alpha: *F*(5) = 0.498, *p* = 0.777, η_p_^2^ = 0.037, power = 0.175; Beta: *F*(5) = 1.901, *p* = 0.106, η_p_^2^ = 0.128, power = 0.609]. Therefore, we did not find any significant differences in the local efficiency across the various phases of the endurance cycling task for any of the analyzed frequency bands.

On the other hand, the within-subjects one-way ANOVA for GE yielded significant differences among the six intervals of the endurance cycling task for the Alpha band [*F*(5) = 3.749, *p* = 0.005, η_p_^2^ = 0.224, power = 0.914; see Figure 3B], but not for the Beta band [*F*(5,2.811) = 2.105, *p* = 0.120, η_p_^2^ = 0.139, power = 0.478; see Figure 3E]. As shown in Figure 3B, *post hoc* pairwise comparisons revealed significant differences between pre-cycling and cycling stage 1 (*p* = 0.005, Cohen *d* = 1.283) and between pre-cycling and cycling stage 2 (*p* = 0.005, Cohen *d* = 1.276), highlighting a significant increase of the global efficiency in the Alpha band during the initial phases of the endurance cycling task.

The within-subjects one-way ANOVA for D values also showed significant differences among the six intervals of the endurance cycling task for the Beta band [*F*(5) = 5.252, *p* < 0.001, η_p_^2^ = 0.288, power = 0.981; see Figure 3F], but not for the Alpha band [*F*(5) = 0.646, *p* = 0.665, η_p_^2^ = 0.047, power = 0.221; see Figure 3C]. *Post hoc* pairwise comparisons showed significant differences between pre-cycling and cycling stage 3 (*p* = 0.047, Cohen *d* = 0.966), between cycling stage 1 and cycling stage 3 (*p* = 0.031, Cohen *d* = 1.024), and between cycling stage 3 and passive recovery (*p* = 0.007, Cohen *d* = 1.239) (see Figure 3F), highlighting a significant decrease of the extent of functional connectivity in the Beta band during the initial phases of the endurance cycling task and a subsequent significant increase of the extent of functional connectivity in the Beta band during the final phases of the endurance cycling task. Table 2 provides the mean and the range for LE, GE, and D found for the six task periods in the Alpha and the Beta bands.

**TABLE 2:** Mean and SEM of local efficiency (LE), global efficiency (GE), and density (D) for each interval of the endurance cycling task in the Alpha and the Beta bands (0 = pre-cycling, 1 = cycling stage 1, 2 = cycling stage 2, 3 = cycling stage 3, 4 = active recovery, and 5 = passive recovery).

Graph indices	Frequency band	Period	Mean	Standard error of mean
LE	Alpha	0	0.578	0.012
		1	0.603	0.028
		2	0.585	0.018
		3	0.575	0.022
		4	0.600	0.024
		5	0.565	0.022
	Beta	0	0.599	0.022
		1	0.660	0.026
		2	0.576	0.024
		3	0.581	0.023
		4	0.605	0.019
		5	0.631	0.021
GE	Alpha	0	0.546	0.011
		1	0.587	0.008
		2	0.588	0.009
		3	0.556	0.008
		4	0.562	0.011
		5	0.555	0.009
	Beta	0	0.574	0.010
		1	0.523	0.018
		2	0.566	0.013
		3	0.557	0.010
		4	0.562	0.014
		5	0.574	0.013
D	Alpha	0	0.263	0.006
		1	0.263	0.004
		2	0.264	0.004
		3	0.255	0.004
		4	0.259	0.006
		5	0.268	0.007
	Beta	0	0.274	0.006
		1	0.273	0.005
		2	0.262	0.003
		3	0.250	0.004
		4	0.260	0.004
		5	0.268	0.004

## Discussion

The aim of this exploratory study was to examine how functional connectivity at the source level (i.e., across brain areas) changed during the course of an endurance cycling task. The connectivity maps were calculated for two meaningful frequency bands (Alpha and Beta), and the properties of the functional networks obtained were quantified using indices derived from the Graph Theory.

We found that LE did not change significantly across the six defined intervals of the endurance cycling task, regardless of the frequency band considered. Given that LE remained essentially constant during the task in both bands (except for a small increase during cycling stage 1 in the Beta band), we can speculate that fatigue processes did not affect segregated neural processing (Rubinov and Sporns, 2010). It is noteworthy that we observed a sparse rather than a local connectivity pattern involving the fronto-parietal areas in each interval of the task (see Figure 2). The lack of significant LE changes during task execution and the corresponding sparse connectivity patterns could be justified by the fact that a cycling task is bilateral in nature, hence requiring sustained movement and effort (Comani et al., 2014). Accordingly, cortico-cortical communication, even during the stages of sustained movement, cannot be considered as locally distributed and efficient. Despite the perception that fatigue might lead to a local reorganization of the cortical networks (Berchicci et al., 2013), our results contradict the notion that an expert athlete (e.g., an athlete who practices at least twice a week) engages in less cortico-cortical communication in the Alpha and the Beta bands (Deeny et al., 2009). Of note is that the patterns of less cortico-cortical communication in the Alpha and the Beta bands were mainly observed in predominantly unilateral and precision sports such as shooting or archery, for which strong contro-lateral cortical patterns were also observed (Deeny et al., 2009). Notwithstanding the lack of significant differences, we observed an increased LE during cycling stage 1 (see Figures 3A,D). This result could be due to the transition from a period of global resting state (i.e., pre-cycling) that requires an extensive exchange of background information among all brain regions before task execution and movement (Petsche et al., 1997; Pfurtscheller and Andrew, 1999) to a period of sustained movement (di Fronso et al., 2018) such as cycling stage 1.

A similar result was obtained for GE. In particular, we observed significant differences on GE values between pre-cycling and cycling stage 1 and between pre-cycling and cycling stage 2, mainly apparent in the Alpha band and characterized by clearly increased values. The increase of GE in the Alpha band during cycling stages 1 and 2 could be due to greater alertness processes and preparedness (Gutmann et al., 2015) to stimuli during exercise (such as the increase of the power level of the cycle-ergometer). Since GE is a measure of functional integration in the brain, which reflects the ability to combine specialized information from distributed brain regions, the increased GE values could also be related to the tendency of central and temporal areas to overlay portions of the sensorimotor cortex that receive afferent feedback during exercise (Dishman et al., 2010). Moreover, the increased metabolic demands and arousal during exercise might have elicited diffuse increases in cortical EEG activity (Steriade et al., 1990).

The GE decrease in cycling stage 3 is partly in line with the findings obtained by di Fronso et al. (2018), who showed reduced coherence during the periods of an exhaustive cycling task characterized by high effort. Indeed during cycling stage 3 our participants were approaching exhaustion and, consequently, their highest perceived sustainable effort (see Table 3). Moreover, a high effort could likely make it harder to integrate information between different brain areas (Kong et al., 2015). In this perspective, it makes sense that the GE values clearly decrease during cycling stage 3 and subsequently increase during active and passive recovery, which are periods characterized by lower effort and absence of movement, respectively. Our results on GE during the pre-cycling period seem to disconfirm the aforementioned extensive exchange of background information among all brain regions before task execution and movement (Petsche et al., 1997; Pfurtscheller and Andrew, 1999; di Fronso et al., 2018). Indeed we noticed lower GE values during pre-cycling as compared to those during movement periods. This finding could be related to the easy nature of the task subsequently performed (i.e., pedaling), which does not involve complex demands (e.g., attentional demands).

**TABLE 3:** Rated perceived exertion (RPE) values for the five task periods during which RPE was collected.

Period	Mean	Standard error mean
Cycling stage 1	1.370	0.235
Cycling stage 2	4.004	0.288
Cycling stage 3	8.576	0.346
Active recovery	4.536	0.487
Passive recovery	1.825	0.403

During active and passive recovery, the restoring of values similar to those of the pre-cycling period is observed also for D, which reflects the extent of connectivity in a network. Indeed we observed—especially in the Beta band—significant differences between cycling stage 3 and passive recovery, with increased values during recovery. These results partly concur with the idea that, after a fatiguing cycling exercise, there is an increase in the communication between the mid/anterior insula and the motor cortex (Hilty et al., 2011). On the other hand, the significant difference obtained between pre-cycling and cycling stage 3 could be interpreted in terms of “efficiency,” that is, the cost of transmitting information within the network: an organization of brain networks, during high effort, may likely approach the maximum possible cost efficiency (Poldrack, 2015). The significant reduction of D during cycling can also be ascribed to the fact that task execution becomes more automated and less controlled during cycling stage 3, despite the high effort due to the increasing power level of the cycle-ergometer. The general reduction of functional connectivity during cycling stage 3 can be interpreted within the framework of the neural efficiency hypothesis (Callan and Naito, 2014), which posits a reduction of nonessential functional connectivity within the brain for expert/automated performances (Hatfield and Kerick, 2007). The decreased values of both GE and D indices during stage 3 may also have been influenced by a decision-making process (e.g., to endure or withdraw from a demanding task) typical of the last stages of an endurance task (Allen et al., 2013). In line with decision making tenets, during cycling stage 3, the connectivity could be more restricted to pre-frontal cortical areas (Kennerley and Walton, 2011).

Some limitations of our investigation should be considered for future research. First, the artifacts deriving from cycling affect low-frequency ranges: for this reason, we did not consider the Theta band in our study. Future studies examining also the Theta band within a graph theory framework could better address the neural efficiency hypothesis. Additionally, examining other graph theoretical indices, such as nodes (ROIs) and/or edges (functional connections between ROIs) betweenness centrality (He et al., 2009; Sporns, 2013, 2018), could enable a better understanding of which ROIs and which connections are most active during the different phases of an endurance cycling task. Also, to better understand how graph indices vary during active and passive recovery, longer periods of post-task recovery should be considered. Finally, to obtain more generalizable findings, the graph theory analysis should be extended to different endurance tasks and to a larger sample of experienced participants. Future studies should also envisage protocols including a control group to better examine patterns of cortico-cortical communications in the Alpha and the Beta bands.

To our knowledge, our study is one of the first ones attempting to estimate functional connectivity at the source level to typify endurance performance. Despite the mentioned limitations, we argue that the use of graph theoretical indices may represent a clear methodological advancement in investigating the brain patterns associated with the different phases of an endurance task and can provide new insights on the neurophysiological correlates of endurance performance.

## Data Availability Statement

The datasets generated for this study will be made available on request to the corresponding author.

## Ethics Statement

The studies involving human participants were reviewed and approved by Ethics Committee of the University “G. d’Annunzio” of Chieti–Pescara (Italy) (Ethical approval Ref. no. 10-21/05/2015). The patients/participants provided their written informed consent to participate in this study.

## Author Contributions

GT and SdF conceived the study. GT, SdF, MB, and SC designed the study. GT and SC developed the methodology. GT and SdF ran the experiments. GT analyzed the data. GT, SdF, CR, MB, and SC wrote, revised, and edited the manuscript. All authors contributed to the article and approved the submitted version.

## Conflict of Interest

The authors declare that the research was conducted in the absence of any commercial or financial relationships that could be construed as a potential conflict of interest.

## Footnotes

1 http://www.uzh.ch/keyinst/loreta.htm
